# Mechanical Behavior of Al-Si10-Mg P-TPMS Structure Fabricated by Selective Laser Melting and a Unified Mathematical Model with Geometrical Parameter

**DOI:** 10.3390/ma16020468

**Published:** 2023-01-04

**Authors:** Xiaonan Zhang, Xiangyu Xie, Yongjing Li, Bin Li, Shilin Yan, Pin Wen

**Affiliations:** 1 Hubei Key Laboratory of Theory and Application of Advanced Materials Mechanics, School of Science, Wuhan University of Technology, Wuhan 430070, China; 2 School of Mechanical Engineering, Wuhan Polytechnic University, Wuhan 430023, China

**Keywords:** triply periodic minimal surface, selective laser melting, mechanical properties, energy absorption, mathematical model

## Abstract

Compared with the traditional lattice structure, the triply periodic minimal surface (TPMS) structure can avoid stress concentration effectively. Here, it is promising in the fields of lightweight and energy absorption. However, the number of structural parameters and mechanical properties of the TPMS structure is plentiful, and the relationship between them is unclassified. In this paper, for the first time, a unified mathematical model was proposed to establish the relationship between TPMS structural design parameters and mechanical properties. Fifteen primitive models were designed by changing the structural parameters (level-set value *C* and thickness *T*) and manufacturing by selective laser melting. The geometric defects and surface quality of the structures were explored by optical microscope and scanning electron microscopy (SEM). The mechanical properties were investigated by quasi-static compression test and finite element simulation. The influence of building direction on structural mechanical behavior (failure mode, stress-strain curve) was studied. The real mechanical properties (Young’s modulus and plateau stress) of the structure could be predicted according to different *C* and *T* combinations. Finally, the energy absorption characteristics were explored. The results showed that when the *C* value is 0.6 in the range of 0–0.6, the energy absorption performance of the structure is at the maximum level.

## 1. Introduction

Cellular material structures (such as honeycomb structure [1,2], sandwich structure [3,4], and lattice structure [5,6,7], i.e.,) are widely used in aerospace [8], automobile manufacturing [9], life science [10,11] and other fields due to lightweight [12,13] and high energy absorbing capacities [14,15]. The design of existing cellular structures is usually inspired by the natural topology structures in the world. Triply periodic minimal surfaces (TPMS) and related materials have been widely found in biological systems like skeletons and scaffolds [16,17]. The gyroid structure is identified under the ribs of butterfly wing scales [16], with a unit cell parameter of ≈300 nm. Besides, the photonic crystal structures with Schwarz-D architectures in the scales of the weevil Lamprocyphus Augustus [17]. It was reported the unit cell parameter was around 450 nm. The TPMS [18,19,20,21] are one class of mathematically defined surfaces that have zero mean curvature. In addition, the TPMS structures are smooth infinite surfaces that partition the space into labyrinths in the absence of self-intersections. Therefore, TPMS structures can effectively avoid stress concentration [7,22,23].

There are two kinds of common TPMS structures: skeleton-based TPMS structures and sheet-based TPMS structures. Al-Ketan et al. [24] explored the mechanical properties of these two different structures under the same density through compression tests and compared them with the strut-based structure. The results showed that the sheet TPMS structure exhibits the best mechanical properties under the same relative density. Zhang et al. [25] studied the mechanical properties and energy absorption abilities of three types of sheet-based TPMS structures (primitive, diamond, and gyroid) fabricated by Selective laser melting (SLM) with 316L stainless steel. The results showed that the mechanical properties of the three types of TPMS structures significantly outperform BCC lattice structures. Among them, the diamond-TPMS structure possesses the best mechanical properties for its smooth shell geometries and stable failure mechanisms. Meanwhile, it is believed that the mechanical properties and deformation mechanism of the TPMS structure are strongly dependent on the unit geometry parameters. Maskery et al. [26] examined three different TPMS structures through compression experiments and simulations. The testing results indicated that TPMS structures, particularly primitive structures, exhibit several potential advantages over lattice structures in terms of load-bearing (higher specific stiffness and strength), energy absorption, etc. The mechanical properties of primitive triply periodic minimal surface (P-TPMS) structures are determined by many factors, such as cell topology, cell size, and thickness [27,28]. However, so far, the effect of cell topology on the structural mechanical behavior of P-TPMS has not been fully understood, and the relational function expression is established without high-throughput experiments.

In this paper, a unified mathematical model between TPMS structural design parameters (level-set *C*, thickness *T*) and mechanical properties is established for the first time. the quasi-static mechanical properties of P-TPMS fabricated by SLM are explored by compression tests and finite element (FE) simulation. The internal defects of the structure are studied. Then the influence of the building direction on the structural mechanical behavior is discussed. Finally, the energy absorption characteristics of the TPMS structure under different design parameters are explored.

## 2. Experimental Details

### 2.1. Primitive Structure Design

Primitive [29], which is a kind of TPMS structure, can be expressed by the following equation:

(1) $$\phi_{p}(x,y,z)=\cos(\omega x)+\cos(\omega y)+\cos(\omega z)=C$$

where $\omega =\frac{2\pi }{l_{i}}$ and $l_{i}$ is the unit cell length in *x, y, z* direction.

**Figure 1:**
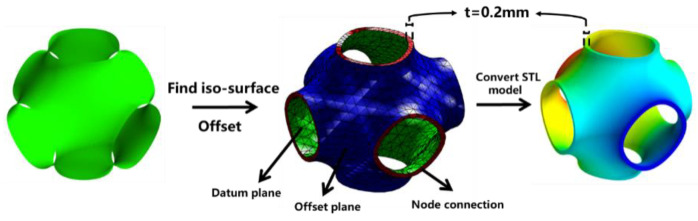
Schematic of P-TPMS structure.

Based on Equation (1), the minimal surface iso-surface is extracted by MATLAB 2019b. The designed surface structure is then shifted along the direction of thickness, and the nodes of the two planes are connected in turn. Finally, a computer-aided design (CAD) model of the P-TPMS structure is obtained. The conversion of the numerical matrix to STL format facilitates the printing of the model and the simulation of structural performance, the main schematic is shown in Figure 1.

**Figure 2:**
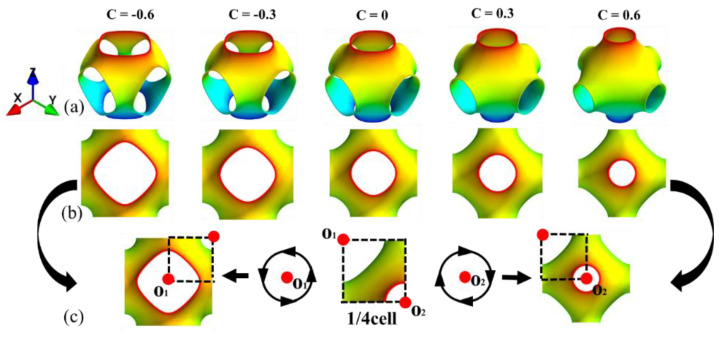
Unit cell geometric configuration of Schwarz’ P surface: (**a**) The 3D schematic of P-TPMS structure; (**b**) the vertical view of P–TPMS structure; (**c**) two formation methods of the surfaces with |*C*|.

The *C* value can only vary from −1~1, otherwise, the surface will disappear. When the absolute value of *C* is close to 1, the holes in the structure will be very small or overlap, which will affect the testing results. Figure 2a shows CAD models of the P-TPMS unit cell of different *C* values. The cell geometry gradually evolves from open-cell to closed one when *C* ranges from −0.6 to 0.6. Meanwhile, the projection of the unit cell in arbitrary planes is shown in Figure 2b. As can be seen, the projection from different directions is the same. When the absolute values |*C*| are equal, a quarter of the surface structure is constrained in a cube, and it is square on the XY projection as shown in Figure 2b.

The unit cells of the primitive surface with *C* = −0.6 and *C* = 0.6 are obtained by rotating 1/8 surface around points O_1_ and O_2_, respectively. Therefore, the quasi-static mechanical response of the P-TPMS lattice structure is explored when the *C* value is positive in this study.

### 2.2. Manufacturing

The P-TPMS samples were manufactured through the SLM process (EP M260, Beijing, China) in the size of 19 mm × 19 mm × 19 mm. The optimized processing parameters for the Al-Si10-Mg power were: laser power of 370 W, hatch spacing of 0.03 mm, scanning speed of 1300 mm/s, and layer thickness of 0.03 μm. The SLM process was performed in the argon atmosphere. The substrate was preheated at 150 °C and this pattern was rotated over 45° between layers. All parts were built using a bidirectional scanning strategy after contour scanning. After printing, the sample was naturally cooled to room temperature in the furnace, then the sample was cut off from the substrate by a wire-cutting process. The samples were soaked in ethanol (analytically pure) for ultrasonic cleaning to remove the adhesive power particles. In order to eliminate the residual thermal stress inside the structure and improve the homogeneity [30,31], all samples were heated with nitrogen protection at 250–300 °C for 2 h followed by cooling in a tube furnace.

**Figure 3:**
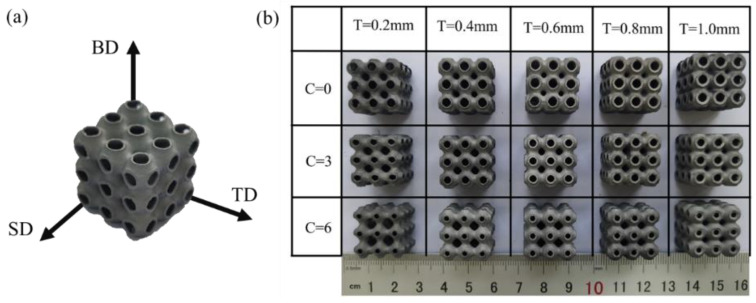
(**a**) Schematic diagram of the scanning strategy. BD represents building direction, SD stands for scanning direction, and TD is the transverse direction. (**b**) Top view of the P-TPMS structure with different structural parameters.

Figure 3a shows the schematic diagram of the P-TPMS structure scanning strategy. By changing the offset thickness and the *C* value, a batch of SLM-printed P-TPMS structures was obtained, as presented in Figure 3b.

### 2.3. Sample Parameters

By using the Archimedes method, the density of the Al-Si10-Mg P-TPMS structure was determined to be 2.7 g/cm^3^. After that, the actual volume of each sample can be calculated by diving its actual weight by density. The theoretical volume of the model was calculated in Abaqus, and the relative density of the structure was obtained by dividing the volume of the structure by the cube volume *V* with the same length, width, and height as the structure. The sample parameters of the P-TPMS structure are presented in Table 1.

The level-set value *C*, the theoretical shell thickness *T*, and the relative density $\bar{\rho }$ measured by the test in Table 1 were simulated with a binary linear equation, the results were as follows:

(2) $${\bar{\rho }}_{exp}=4.7881C+39.0988T$$

**Figure 4:**
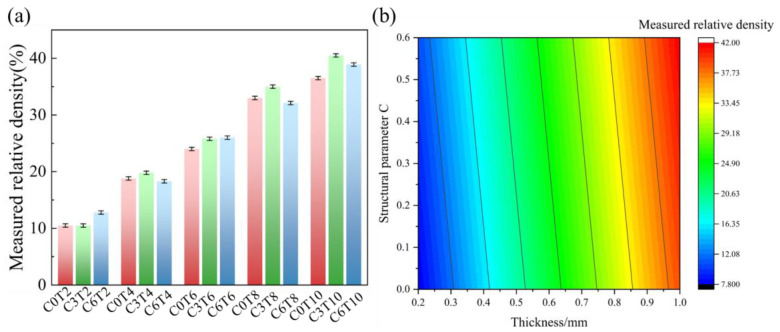
(**a**) The relative density of as-built P-TPMS. (**b**) The predicted value of relative density under different structural parameters.

The *F*-value of the model is 1199.59, and the *p*-value is less than 0.001. The correlation coefficient *R^2^* of the model is 0.994, which proves that the regression effect of the model is significant. According to this empirical formula, the relative density of the as-built Al-Si10-Mg P-TPMS structure under different *C* and *T* parameter combinations can be predicted, as shown in Figure 4.

### 2.4. Compression Performance Test

The compression test was carried out according to ISO13314:2011 [32] in the universal testing machine (SANS CMT5205, Shenzhen, China). To ensure uniform loading and eliminate torque caused by specimen misalignment, the specimen was placed in the center of the loading rack. The deformation behaviors of testing samples were recorded via a digital camera during the compression test. The compression test was performed with a constant velocity (2 mm/min) and the experiment was stopped when the structure reaches densification.

### 2.5. Finite Element Modelling

**Figure 5:**
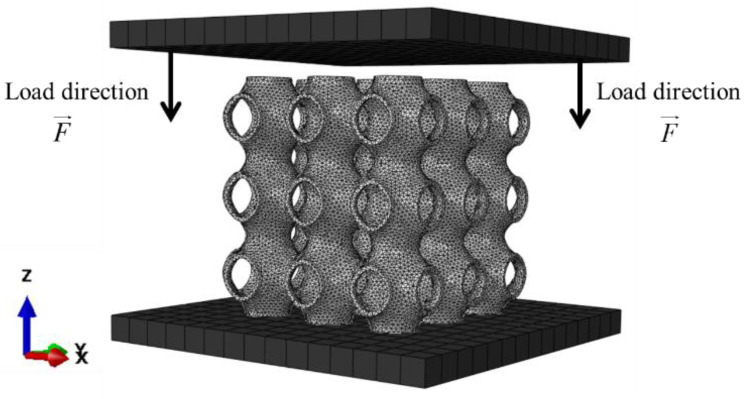
FE model of P-TPMS subjected to quasi-static compression.

The FE simulation was performed in Abaqus 2020, as shown in Figure 5. The primitive structure was placed between two rigid plates. The top plate could only move along the Z-direction, and other degrees of freedom were all fixed. Meanwhile, all the freedom degrees of the bottom plate were fixed as well. The base material Al-Si10-Mg was assumed to be linearly elastic and perfect plastic, the Young’s modulus, yield stress, and Poisson’s ratio were measured to be 70 GPa, 256 MPa, and 0.3, respectively. The general contact was defined between the rigid plates and the primitive structure. To prevent relative slippage between the rigid plates and the primitive structure, the friction coefficient was set to 0.3. The dynamic explicit was applied in the FE due to large deformation problems during structural compression [33]. The primitive structure was modeled with C3D6 elements (a linear triangular prism) with approximately 200,000 elements. Meanwhile, the rigid plates were modeled with R3D4 elements (four nodes 3D bilinear rigid quadrilateral) with an average element size of 0.2 mm.

## 3. Results and Discussion

### 3.1. Structural Defects Induced by SLM

**Figure 6:**
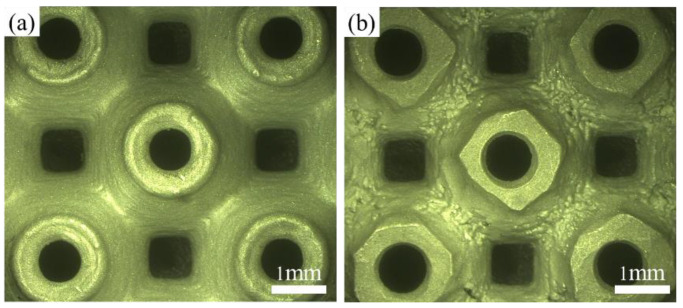
Structure surface under the optical microscope: (**a**) Top of as-built P-TPMS; (**b**) bottom of as-built P-TPMS.

**Figure 7:**
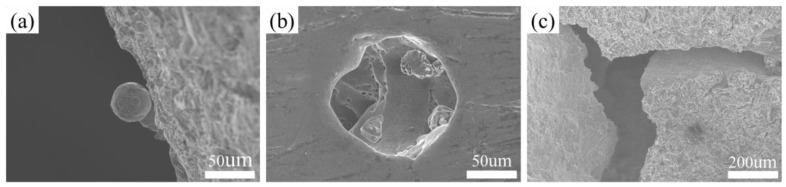
SEM observed the surface morphology of SLM as-built primitive specimen: (**a**) The powder attached to the surface of the structure; (**b**) the holes and unmelted particles; (**c**) the fracture that appears during compression.

Figure 6a,b shows the top and bottom views of the C0T4 P-TPMS structure sample under the optical microscope. The top surface shows smoother than the bottom surface. During the printing process, there is no solid structure support at the bottom, the sample is only supported by the powder bed. Therefore, the powder at the bottom of the structure accumulates and cannot be eliminated in time, which causes an interaction between the solidified structure and the powder layer. This interaction usually leads to the partial melting of the loose powder under the structure, resulting in more surface roughness at the bottom of the structure. Especially when laser scanning and sintering profiles, some of the powder particles are partially melted and combined with the bottom profile layer, which may be the main reason for the irregular bottom structure profile. However, the deviation between the sample print thickness and the theoretical model is measured within 0.03 mm, which indicates the high quality of the printed sample. The scanning electron microscopy (SEM) images of the C0T4 sample are given in Figure 7a, and scattered powder is found to be attached to the surface. In the SLM process, there is a heat-affected zone around the molten pool when laser scanning metal powder, which makes the loose powder around the molten pool adhere. This may be the main reason that the sample printing thickness is larger than the theoretical design. The structure is cut into three layers along the building direction by the wire-cutting process. The holes and unmelted particles of the middle layer in the structure are observed by SEM, as shown in Figure 7b. Figure 7c shows a unit cell selected during structural compression, indicating that brittle fracture will occur during structural compression. These defects may affect the mechanical behavior of structures.

### 3.2. Deformation Mode

**Figure 8:**
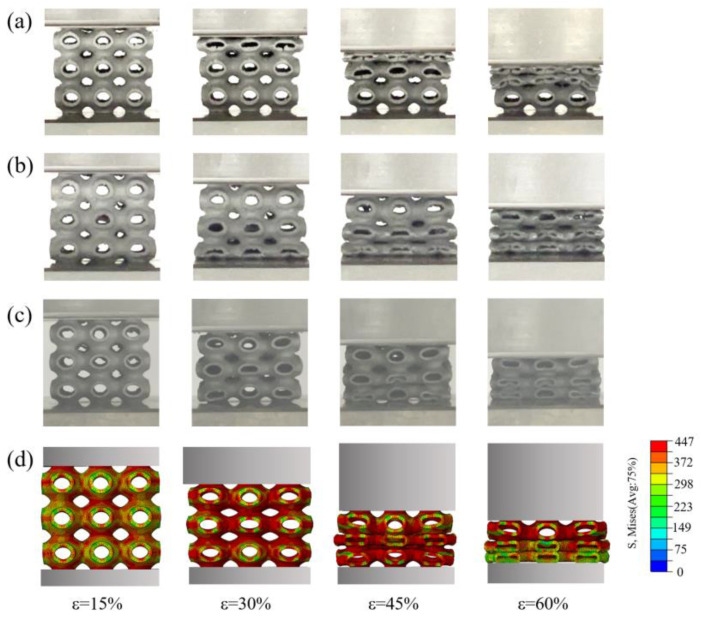
Deformation mode related to building direction: (**a**) The compression direction is consistent with the building direction, $\vec{F}=\vec{B}$; (**b**) the compression direction is opposite to the building direction, $\vec{F}=-\vec{B}$; (**c**) the compression direction is perpendicular to the building direction, $\vec{F}⊥\vec{B}$; (**d**) finite element simulation deformation diagram.

Due to the unavoidable structural defects induced by SLM such as powder adhesion, residual thermal stress, and internal holes in the SLM printing process, the as-built structure has an irregular profile on the bottom of the structure, showing a greater roughness than the top of the structure. Figure 8 shows the deformation mode of the C0T6 P-TPMS structure related to the building direction. When the loading direction is the same as the building direction (a), the structure will collapse layer by layer from top to bottom during the compression process. When the loading direction of the structure is opposite to the building direction (b), the structure will collapse layer by layer from bottom to top. The phenomenon indicates the inhomogeneous mechanical properties of the structure along the building direction. To avoid the influence of this kind of inhomogeneity, the loading direction is set to be perpendicular to the building direction. In this case, the deformation of the structure appears to be more symmetrical. The middle layer crushes first, with the upper and bottom layers to follow. The FE simulation deformation mode diagram of the structure is shown in Figure 8d, which is consistent with the deformation behavior of the structure when the building direction is perpendicular to the loading direction.

When the strain is 15%, the stress distribution and deformation of the upper, middle, and lower layers of the structure are the same due to the symmetry of the structure and boundary conditions. As the strain increases, the upper and lower cells begin to rotate due to the lack of interface constraints. However, due to the constraint of symmetry conditions, the middle cell always shows compression deformation in the loading direction. When the strain reaches 30%, the stress in the middle cell is slightly larger than that in the upper and lower layers, resulting in greater compression deformation of the middle cell. When the strain approaches 45%, the single cells in the middle layer are crushed first. In the experiment, some cells show a brittle failure due to the defects caused by the printing process. When the strain is 60%, the upper, middle, and lower layers of the structure were crushed, and the surface of the structure begins to contact each other and enters the compaction stage. The deformation behavior of the structure in the finite element simulation is in good agreement with the test.

**Figure 9:**
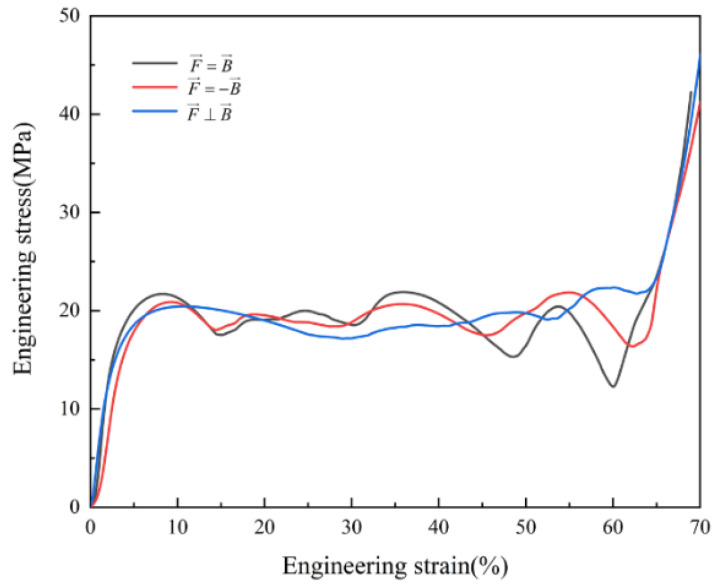
Stress–strain curve related to building direction: The compression direction is consistent with the construction direction, $\vec{F}=\vec{B}$; the compression direction is opposite to the construction direction, $\vec{F}=-\vec{B}$; the compression direction is perpendicular to the construction direction, $\vec{F}⊥\vec{B}$.

Figure 9 shows the relative stress–strain curve of the C0T6 P-TPMS structure. When the loading direction is perpendicular to the construction direction, the stress platform stage of the structure is more stable, while the other two compression modes show greater stress fluctuations. When the strain is 10%, 35%, and 55%, the stress–strain curve drops prominently, indicating that the building direction will have a very significant impact on the mechanical behavior of the structure. Therefore, this phenomenon can be avoided by letting the loading direction be perpendicular to the building direction during the placement of the structure.

### 3.3. Stress–Strain Curve

**Figure 10:**
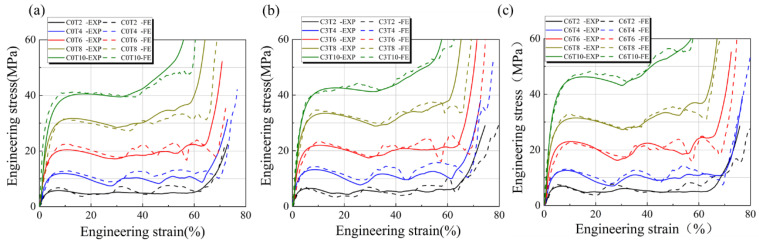
The strain–stress curve of P-TPMS structures in experiment and FE simulation. (**a**) Shows as-built samples with *C* values of 0 and different thickness of 0.2, 0.4, 0.6, 0.8 and 1.0mm. (**b**) Shows as-built samples with *C* values of 0.3 and different thickness of 0.2, 0.4, 0.6, 0.8 and 1.0mm. (**c**) Shows as-built samples with *C* values of 0.6 and different thickness of 0.2, 0.4, 0.6, 0.8 and 1.0mm.

The stress and strain curves of the P-TPMS structure with different C values are obtained through FE analysis and compression test, as shown in Figure 10.

The predicted value obtained by FE simulation is found to be in good agreement with the experimental result. The slight deviation can be attributed to the powder adhesion, a large molten pool, and the existence of residual stress. In addition, there are holes and other defects in the structure and brittle fractures will occur during the compression process, which is different from the ideal elastic model in FE simulation. The development of the compression of the P-TPMS structure is as follows: in the first stage, such as the linear elastic stage, the stress increases linearly with the strain value. When the stress–strain curve deviates from linearity to nonlinearity, the stress increases continuously from the second stage to the peak of the maximum stress. At the peak stress, the sample shows obvious deformation, and then the stress–strain curve will fluctuate within a certain range, which may be caused by the existence of holes and unmelted particles in the structure. Finally, the structure reaches the densification stage, and the stress increases linearly with the increase in strain. This is because the surfaces of the structure begin to contact each other, and the porous structure is pressed into a block. Therefore, this stage is also known as the structural failure stage. In addition, the stress–strain curve of the compression test begins to decrease gradually after the strain reaches the highest point at about 5%, indicating that the internal surface of the lattice structure began to break or the local collapse of the structure occurred. With the increase in volume fraction, the strain value corresponding to the yield stage of the structure tends to increase, indicating that the overall plasticity and toughness of the lattice structure will increase with the relative density of the structure.

### 3.4. Relationship between Structural Parameters and Mechanical Properties

The Gibson–Ashby model [34] can be used to describe the influence of relative density on mechanical properties and predict the mechanical parameters of primitive lattice structures with different volume fractions, such as Young’s modulus and plateau stress, as follows

(3) $$\frac{E}{E_{s}}=a{\bar{\rho }}^{m}$$

(4) $$\frac{\sigma_{pl}}{\sigma_{s}}=b{\bar{\rho }}^{n}$$

where E is the young’s modulus of the lattice structure, $E_{s}$ is the young’s modulus of the base material, $\sigma_{pl}$ is the plateau stress, and $\sigma_{s}$ is the yield strength. The elastic modulus is determined by the slope of the linear part of the stress–strain curve. According to ISO 13314:2011 [32], cellular structure yield strength (plateau stress) is defined as the arithmetic mean value of 20–30% or 20–40% stress in the strain range.

(5) $$\sigma_{pl}=\frac{1}{\varepsilon_{x_{1}}-\varepsilon_{x_{2}}}\int_{\varepsilon_{x_{2}}}^{\varepsilon_{x_{1}}}\sigma \left(\varepsilon \right)d\varepsilon$$

The experimental results can be used to simulate the nonlinear equation and predict the mechanical response of primitive structures under different volume fractions. The functional relationship between relative Young’s modulus $E/E_{s}$, relative plateau stress $\sigma_{pl}/\sigma_{s}$ and relative density $\bar{\rho }$ can be fitted by the test data. The fitting nonlinear function is:

(6) $$E/E_{S}=0.0098{\bar{\rho }}^{1.3669}$$

(7) $$\sigma_{pl}/\sigma_{s}=0.0001{\bar{\rho }}^{1.9618}$$

**Figure 11:**
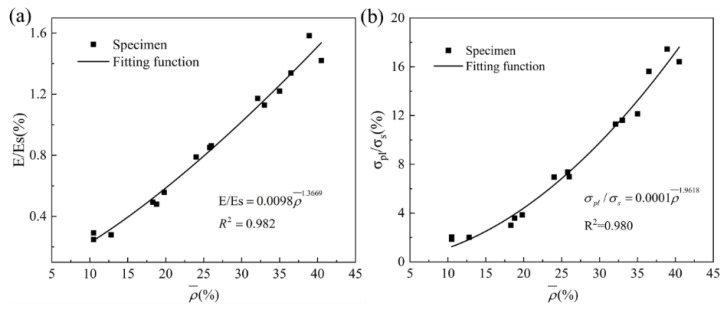
The relative mechanical properties with respect to the relative density: (**a**) Young’s modulus; (**b**) plateau stress.

Fitting coefficients *R^2^* are 0.982 and 0.980, indicating the adaptation precision is high, as shown in Figure 11a,b.

To establish the relationship between structural parameters and mechanical properties of TMPS, together with Equation (2) and Equations (6) and (7), we have

(8) $$E/E_{s}=0.0098(4.7881C+39.0988t{)}^{1.3669}$$

(9) $$\sigma_{pl}/\sigma_{s}=0.0001(4.7881C+39.0988t{)}^{1.9618}$$

Equations (8) and (9) establishes a unified mathematical model between the minimal surface design parameters (level-set parameter *C*, thickness *T*) and Young’s modulus, plateau stress. The real mechanical properties of the Al-Si10-Mg primitive TPMS structure fabricated by SLM can be directly predicted through the structural parameters (*C*, *T*).

### 3.5. Energy Absorption

Specific energy absorption (SEA) is a key index to evaluate the energy absorption capacity of cellular materials. It is defined as the ratio of total energy absorption to mass, and the formula is as follows:

(10) $$SEA=\frac{\int_{0}^{l}F\left(x\right)dx}{m}=\frac{V\int_{0}^{\varepsilon_{d}}\sigma \left(\varepsilon \right)d\varepsilon }{m}$$

where *m* represents the mass of the sample and $\varepsilon_{d}$ is the dense strain of the cellular structure, which can be determined by the energy absorption efficiency [35,36]. The specific method is given as follows: based on the uniaxial stress–strain curve of the lattice structure, the energy absorption efficiency can be calculated as

(11) $$\eta \left(\varepsilon \right)=\frac{1}{\sigma \left(\varepsilon \right)}\int_{0}^{\varepsilon }\sigma \left(\varepsilon \right)d\varepsilon$$

The strain at which the energy absorption efficiency reaches the maximum on the efficiency strain curve is defined as the initial densification strain

(12) $${\frac{d\eta \left(\varepsilon \right)}{d\varepsilon }|}_{\varepsilon =\varepsilon_{d}}=0$$

**Figure 12:**
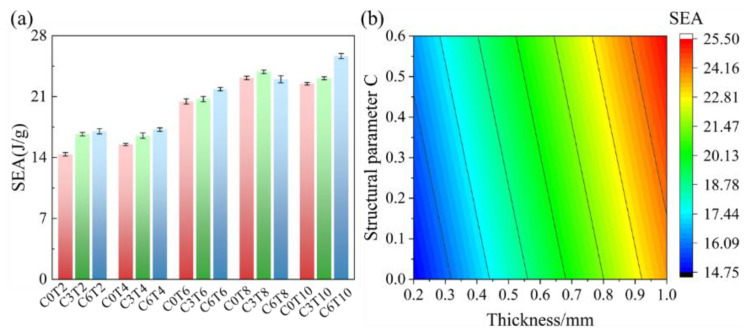
(**a**) Specific energy absorption of as-built primitive TPMS. (**b**) The predicted value of specific energy absorption under different structural parameters.

Figure 12a shows the specific energy absorption diagram of the P-TPMS lattice structure of different structural parameters. The result shows that the size of structural cells will change the energy absorption performance of the structure. Under the same thickness, the energy absorption performance of the structure will be improved to a certain extent with the increase in the level set constant *C*. *C6T10* structure has the best energy absorption performance, reaching 25.654 J/g. When the thickness *T* = 0.2 mm and *T* = 0.4 mm, the energy absorption performance of the structure changes slightly. When the thickness *T* increases from 0.6 mm to 1 mm, the energy absorption performance of the structure is significantly improved.

The specific energy absorption of P-TPMS in Figure 12 is simulated with the level-set value *C* and the theoretical shell thickness *T*. The results are as follows:

(13) SEA=2.930528C+11.17847T+12.51714

According to this empirical formula, the energy absorption performance of the Al-Si10-Mg P-TPMS structure under different *C* and *T* parameters combinations can be predicted, as shown in Figure 12b.

## 4. Conclusions

This paper studies the quasi-static mechanical properties of Al-Si10-Mg TPMS structures fabricated by SLM by compression tests and FE simulation. The structural defects and the effect of the building direction on the structural mechanical behavior (failure mode, stress–strain curve) are investigated by optical microscope and SEM. The mechanical properties of P-TPMS structures under different structural parameters are obtained. The main conclusions are drawn as follows:

1. In the SLM process of the primitive structure, the print quality of the bottom surface is inferior to the top, which results in the inhomogeneity of the mechanical properties along the building direction. It is easily ignored when testing the mechanical properties of 3D-printed specimens. The loading direction is parallel to the building direction, the deformation mode will become crushed layer by layer along the building direction.
2. However, when the loading direction is perpendicular to the building direction, the as-built primitive structure presents symmetrical buckling and has more stable plateau stress.
3. The compression deformation mode of primitive structure can be divided into three stages: linear elastic stage, plateau stage, and densification stage. The thicker the structure thickness in the range of 0.2–1.0 mm, the longer the elastic deformation stage and the shorter the stress platform. The greater the value of *C* in the range of 0–0.6, the greater the fluctuation of the stress platform of the structure.
4. A unified mathematical model between minimal surface design parameters and Young’s modulus, plateau stress is established, which can directly predict the real mechanical properties of Al-Si10-Mg primitive TPMS structure fabricated by SLM through structural parameters (*C*, *T*).
5. When the *C* value is 0–0.6 and the thickness *T* is 0.2–1.0mm, the specific energy absorption of the primitive structure is 14.37–25.65 J/g. Under the same thickness, properly increasing the *C* value can improve the energy absorption performance of the structure. Meanwhile, a mathematical model is established to predict the energy absorption performance of structures of different parameters.

## Figures and Tables

**Table 1:** Details of the primitive structures studied in this paper.

Sample	Level-Set Value	Nominal Shell Thickness (mm)	Measured Shell Thickness (mm)	Nominal Relative Density (%)	Measured Relative Density (%)
C0T2	0	0.2	0.21 ± 0.01	7.4	10.5 ± 0.3
C0T4	0	0.4	0.42 ± 0.02	14.7	18.8 ± 0.3
C0T6	0	0.6	0.62 ± 0.01	21.7	24.0 ± 0.3
C0T8	0	0.8	0.83 ± 0.02	28.4	33.0 ± 0.3
C0T10	0	1.0	1.03 ± 0.02	34.5	36.5 ± 0.3
C3T2	0.3	0.2	0.22 ± 0.01	7.4	10.5 ± 0.3
C3T4	0.3	0.4	0.42 ± 0.02	14.9	19.8 ± 0.3
C3T6	0.3	0.6	0.63 ± 0.02	22.3	25.8 ± 0.3
C3T8	0.3	0.8	0.82 ± 0.02	29.5	35.0 ± 0.3
C3T10	0.3	1.0	1.04 ± 0.03	36.4	40.5 ± 0.3
C6T2	0.6	0.2	0.23 ± 0.02	7.1	12.8 ± 0.3
C6T4	0.6	0.4	0.42 ± 0.01	14.6	18.3 ± 0.3
C6T6	0.6	0.6	0.63 ± 0.02	22.3	26.0 ± 0.3
C6T8	0.6	0.8	0.84 ± 0.02	30.0	32.1 ± 0.3
C6T10	0.6	1.0	1.04 ± 0.02	37.6	38.9 ± 0.3

## Data Availability

Data will be made available on request.
